# Supplementary material for: MoCoLo: a testing framework for motif co-localization
Source: Brief Bioinform. 2024 Feb 1;25(2):bbae019. doi: 10.1093/bib/bbae019 (PMC10960634; doi:10.1093/bib/bbae019)
Supplement: MoCoLo-Supplement_bbae019 [file mocolo-supplement_bbae019.docx]

**Supplementary information for**

**MoCoLo: a testing framework for motif co-localization**

Qi Xu^1,2^, Imee M.A. del Mundo^3^, Maha Zewail-Foote^4^, Brian T. Luke^5^, Karen M. Vasquez^3*^,

Jeanne Kowalski^2*^

^1^Department of Molecular Biosciences, College of Natural Sciences, University of Texas at Austin, Austin, TX, 78712

^2^Department of Oncology, Dell Medical School, University of Texas at Austin, Austin, TX, 78712

^3^Dell Pediatric Research Institute, Division of Pharmacology and Toxicology, College of Pharmacy, The University of Texas at Austin; Austin, Texas, 78723, USA.

^4^Department of Chemistry and Biochemistry, Southwestern University, Georgetown, TX, 78626, USA

^5^Bioinformatics and Computational Science, Frederick National Laboratory for Cancer Research, Frederick, Maryland, 21701, USA

*Co-corresponding/senior authors.

Email: Jeanne.Kowalski@austin.utexas.edu and karen.vasquez@austin.utexas.edu

**Supplementary Figures**

**Supplementary Figure 1. Comparative Distribution of Overlapped 8-oxo-dG and Non-B Motifs**

**Supplementary Figure 2. The distribution of feature lengths and their overlapped region lengths.**

**Supplementary Tables**

**Supplementary Table 1. The number of overlapped 8-oxo-dG motifs in non-B and the number of non-B motif in 8-oxo-dG from the observed group.**


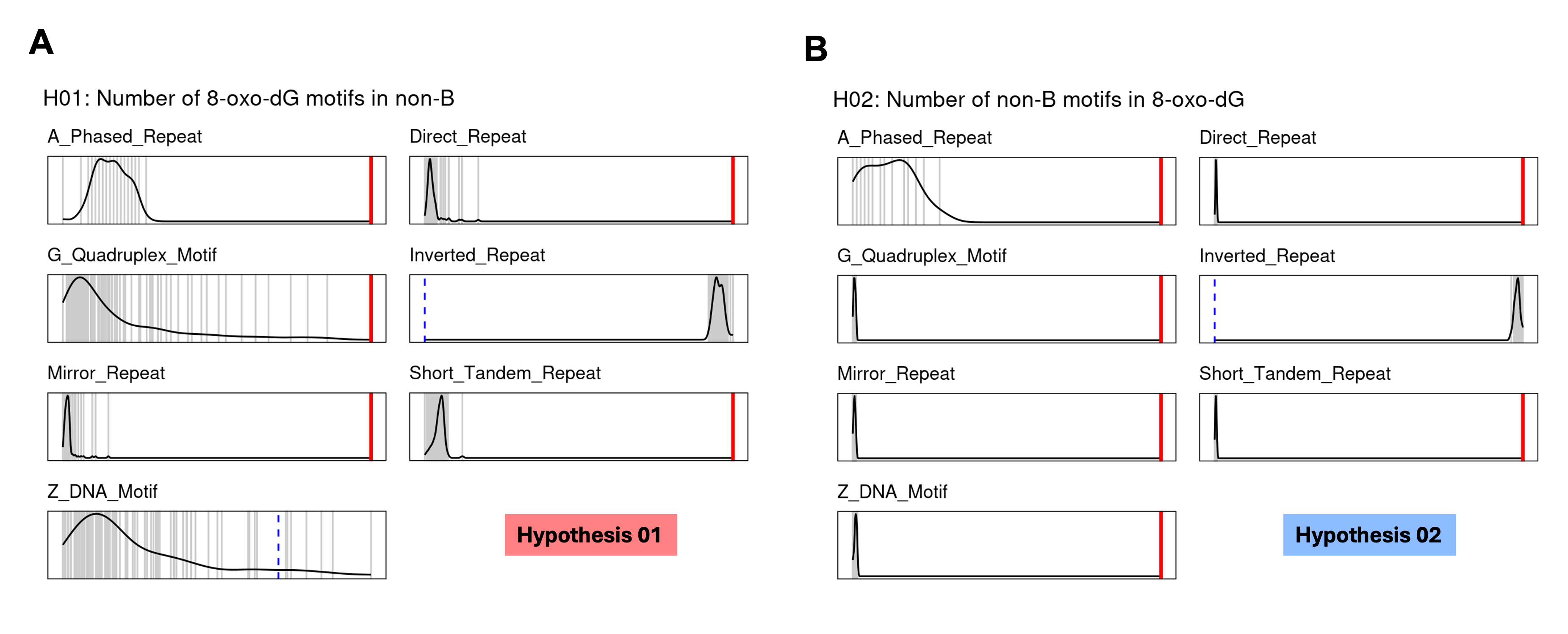


**Supplementary Figure 1. Comparative Distribution of Overlapped 8-oxo-dG and Non-B Motifs**

(**A**) The distribution of 8-oxo-dG motifs within non-B structures, categorized by 7 distinct non-B types in the simulation group (depicted by grey vertical lines, n=100). Corresponding data from the actual group are overlaid with colored lines, where red denotes statistical significance, and blue indicates a lack of significance.

(**B**) The distribution of non-B motifs within 8-oxo-dG structures in the simulation group (depicted by grey vertical lines, n=100). Similarly, overlaying colored lines represent equivalent data from the actual group, with red signifying statistical significance, and blue representing non-significance.

**Supplementary information**


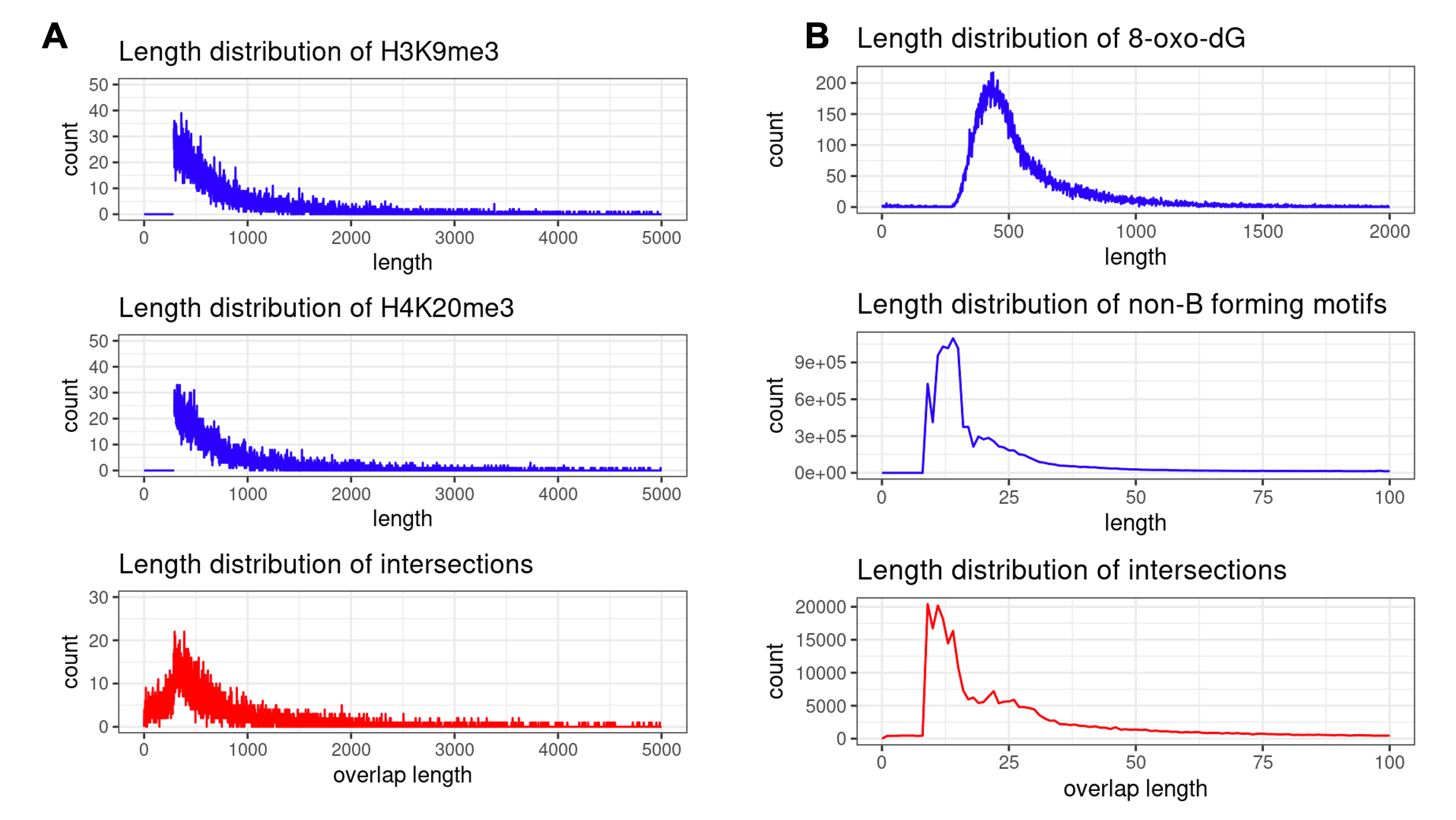


**Supplementary Figure 2. The distribution of feature lengths and their overlapped region lengths.**(**A**) The lengths of H3K9me3 and H4K20me3 peak regions (blue). The lengths distribution of the intersected regions of two features.
(**B**) The lengths of 8-oxo-dG peak regions and all non-B forming motifs (blue). The length distribution of the intersected regions between 8-oxoG and non-B motifs.

**Supplementary information**

**Supplementary Table 1.** The number of overlapped 8-oxo-dG motifs in non-B and the number of non-B motif in 8-oxo-dG from the observed group.
